# R&D Costs of New Medicines: A Landscape Analysis

**DOI:** 10.3389/fmed.2021.760762

**Published:** 2021-10-26

**Authors:** Steven Simoens, Isabelle Huys

**Affiliations:** Department of Pharmaceutical and Pharmacological Sciences, KU Leuven, Leuven, Belgium

**Keywords:** research and development, costs, estimates, medicines, drivers

## Abstract

Over the years, questions have been raised over R&D costs of new medicines. The aim of this study is to conduct a landscape review of the (drivers of) R&D costs of a new medicine derived from the peer-reviewed and grey literature. Included studies have drawn data either from confidential company surveys or from publicly available company financial statements, in addition to accessing the literature and medicine information databases. Although there were differences in methodology, parameter values, samples and time periods between studies, estimates of R&D costs per new medicine (accounting for the cost of failures) ranged from US$944m to US$2,826m (adjusted to 2019 prices). The evidence also suggested that R&D costs per new medicine have increased over time. A few studies have broken down total costs and showed that clinical development accounts for 50-58% of R&D costs per new medicine. R&D costs were influenced by costs of discovery and pre-clinical development, costs of clinical development, cost of capital, company and product profile. Finally, cost estimates are likely to be dynamic as the biopharmaceutical industry and the broader environment continue to evolve.

## Introduction

There has been a long-standing interest in and discussion about the costs to research and develop a new medicine. It is useful to gain insight into those costs as such information may serve to get an idea of how much investment is required to research and develop a new medicine, to use in the assessment of R&D productivity of the biopharmaceutical industry, to inform the debate about medicine prices, or to explain trends in R&D approaches and in the industrial landscape (1, 2).

In the literature, two reviews were published around a decade ago, but these reviews did not include more up-to-date empirical studies estimating R&D costs of a new medicine (2, 3). Recently, a systematic review mainly analysed the relevant peer-reviewed literature, but did not consider consultancy reports on the topic (4). Therefore, the aim of this study is to conduct a landscape review of the costs of R&D of a new medicine derived from the peer-reviewed and grey literature. Also, this study identifies which factors drive R&D costs, based on how such costs were generally calculated in empirical studies.

## Methods

A search was carried out in PubMed to identify studies which estimated R&D costs (and their components) of new medicines based on empirical data. Articles that commented on R&D costs, but did not provide new estimates were excluded. Studies were considered if they had been published between 2000 and 2020, as earlier cost estimates are unlikely to reflect current R&D processes. The search was restricted to English-language studies. Also, the grey literature was searched in Google (Scholar), including empirical studies conducted by consultancy agencies (e.g., Deloitte, Office of Health Economics). Finally, the snowballing method was applied, identifying additional studies from the bibliography of included studies.

A data extraction sheet compiling information about the objectives, design, results, conclusions and limitations was completed for each included study. For the purpose of comparing results between studies, all cost estimates were adjusted to 2019 prices using the World Bank GDP deflator (5).

## Results

Our search of the peer-reviewed literature identified three literature reviews (cfr. supra), eight empirical studies on R&D costs of a new medicine which derived their data from confidential company surveys in addition to the literature and medicine information databases, and two empirical studies which derived their data from publicly available company financial statements. The grey literature generated a series of Deloitte reports on R&D costs based on company financial statements. Another consultancy report by Bain and Company provided cost estimates, but was excluded because no details of the underlying methodology were reported (6). Data extraction sheets for these studies can be found in the Supplementary Material and the results are examined in the following sections.

### Cost Estimates Based on Company Surveys

In 2003, a study calculated the pre-tax R&D costs per new medicine (accounting for the cost of medicine failures) based on a random sample of 68 medicines (both chemical and biologic medicines) developed in-house by 10 multinational companies (1). This study provided the basis for further research. The authors subsequently calculated costs for different product classes (7) and generated a separate estimate for biotechnology medicines (8). Other researchers have attempted to validate the 2003 cost estimate by extracting some parameter values from sources other than company surveys, by using different (methods to calculate) parameter values, or by drawing on a different sample of medicines (2, 9–12). The 2003 research team updated their cost estimate based on a larger sample of 106 in-house developed medicines in 2016 (13).

**Figure 1:**
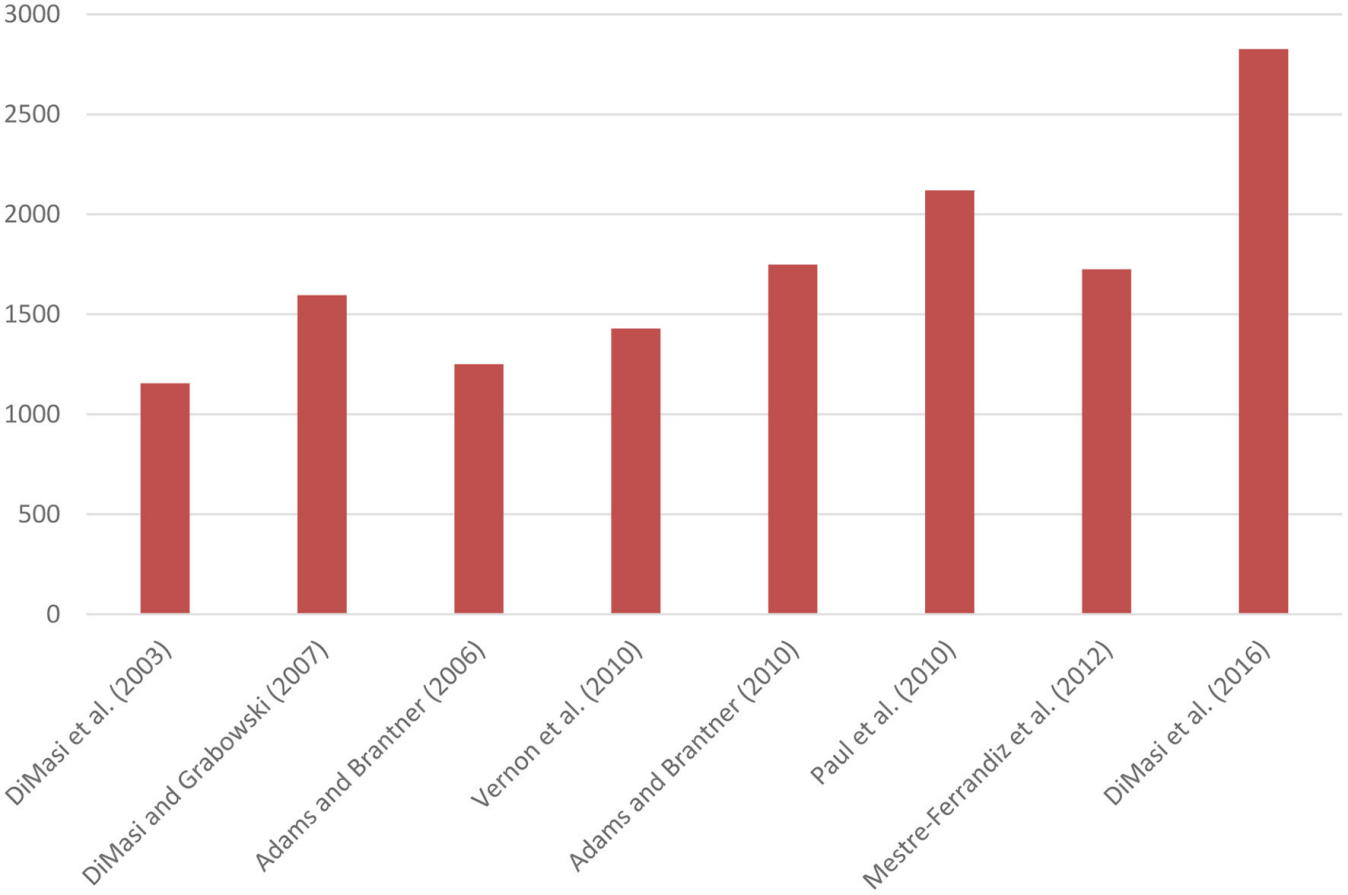
Mean capitalised R&D costs per new medicine based on company surveys (expressed in 2019 US$ millions). DiMasi and Grabowski (8) selected a sub-sample of biotechnology medicines from DiMasi et al. (1).

Figure 1 presents mean capitalised R&D costs per new medicine (adjusted to 2019 prices) of studies based on company surveys. Mean capitalised costs per new medicine amounted to US$1,155m according to the 2003 study (1). When costs of post-marketing authorisation R&D were included, costs per new medicine increased to US$1,292m. The authors also showed that mean capitalised costs per new medicine were similar for chemical and biologic medicines (8). Other studies estimated mean capitalised costs per new medicine to range from US$1,250m to US$2,120m (2, 9–12). The study published in 2016 found that mean capitalised costs per new medicine were US$2,826m (or US$3,171m including costs of post-marketing authorisation R&D) (13).

Although there are differences in methodology, parameter values, samples and time periods between studies, Figure 1 suggests that pre-tax R&D costs per new medicine have increased over time, as also corroborated by a previous literature review (3). Two studies also demonstrated that costs varied between product classes (7, 9).

**Figure 2:**
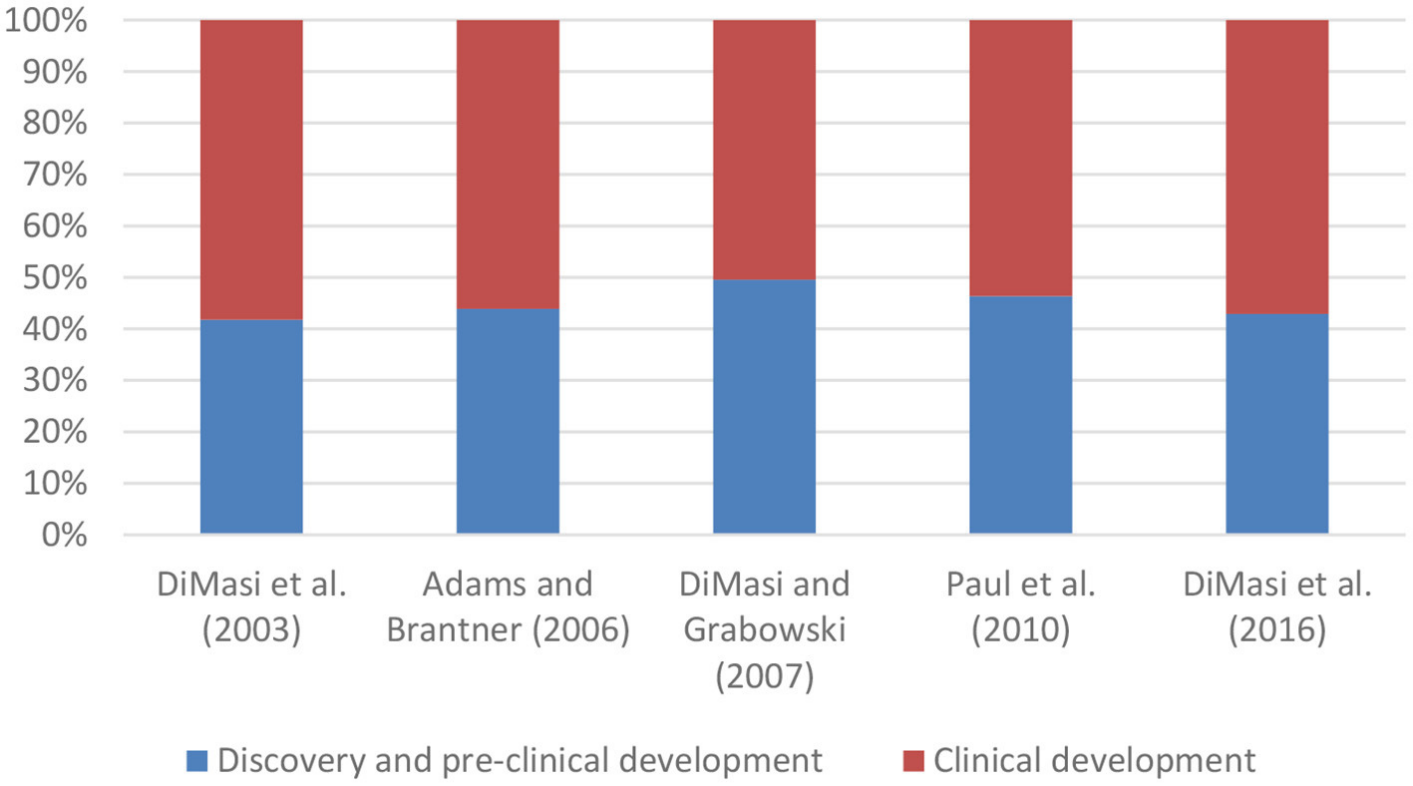
Composition of mean capitalised R&D costs per new medicine. DiMasi and Grabowski (8) selected a sub-sample of biotechnology medicines from DiMasi et al. (1).

A few studies have also broken down mean capitalised R&D costs per new medicine into costs for discovery and pre-clinical development and into costs for clinical development (see Figure 2). These studies showed that clinical development accounts for 50-58% of total costs.

### Cost Estimates Based on Company Financial Statements

Two more recent studies have calculated the pre-tax R&D costs per new medicine (accounting for the cost of medicine failures) based on publicly available company financial statements, thus allowing to validate and replicate their cost estimates. Both studies included in-house developed medicines as well as acquired medicines. One study focused specifically on ten oncology medicines (nine of which had orphan medicine designation) from small companies (14). The other study enrolled a sample of 63 medicines developed by 47 companies, but medicines with specific characteristics (i.e., orphan medicines, medicines in some disease areas, first-in-class medicines, medicines with expedited marketing authorisation, medicines approved by the US Food and Drug Administration between 2014 and 2018, medicines from smaller companies) were over-sampled (15).

The study focusing on oncology medicines found that mean capitalised R&D costs per new medicine amounted to US$944m (adjusted to 2019 prices) (14). Also, costs were significantly higher for in-house developed medicines than for acquired medicines. The other study generated an estimate of mean capitalised R&D costs per new medicine of US$1,359m (15). When limiting the sample to 23 medicines for which high-quality data were available, mean capitalised R&D costs per new medicine decreased to US$1,163m. Additionally, this study documented cost differences between product classes.

Based on the financial statements of the 12 largest biopharmaceutical companies (in terms of R&D investment in 2009), public sources and medicine information databases, Deloitte has calculated R&D costs per new medicine (accounting for the cost of medicine failures) since 2010 (16). Additionally, the analysis was extended to include four smaller, more specialised biopharmaceutical companies since 2013. The sample consisted of new chemical medicines, biologic medicines, line extensions, reformulations, fixed dose combinations and biosimilars that were developed in-house, that were acquired or that were co-developed.

**Figure 3:**
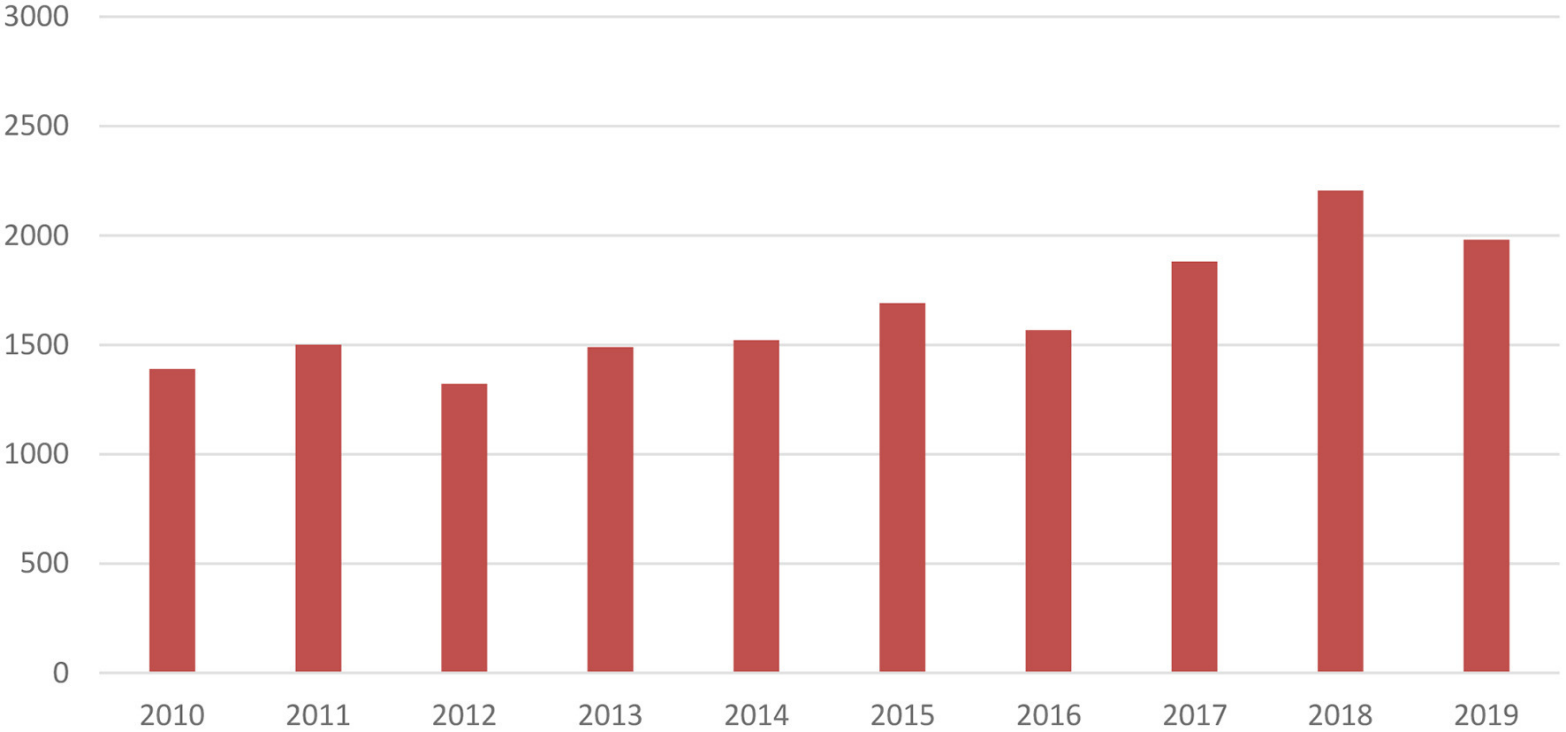
Mean R&D costs per new medicine of 12 largest biopharmaceutical companies over time (expressed in 2019 US$ millions) (16).

As visualised in Figure 3 and adjusted to 2019 prices, mean R&D costs per new medicine of the 12 largest biopharmaceutical companies have increased from US$1,390m in 2010 to US$1,981m in 2019 (+43%). Focusing on the four smaller, more specialised biopharmaceutical companies, a more substantial increase in mean costs per new medicine by 112% was observed from US$1,142m in 2013 to US$2,422m in 2019.

### Drivers of R&D Costs of New Medicines

**Figure 4:**
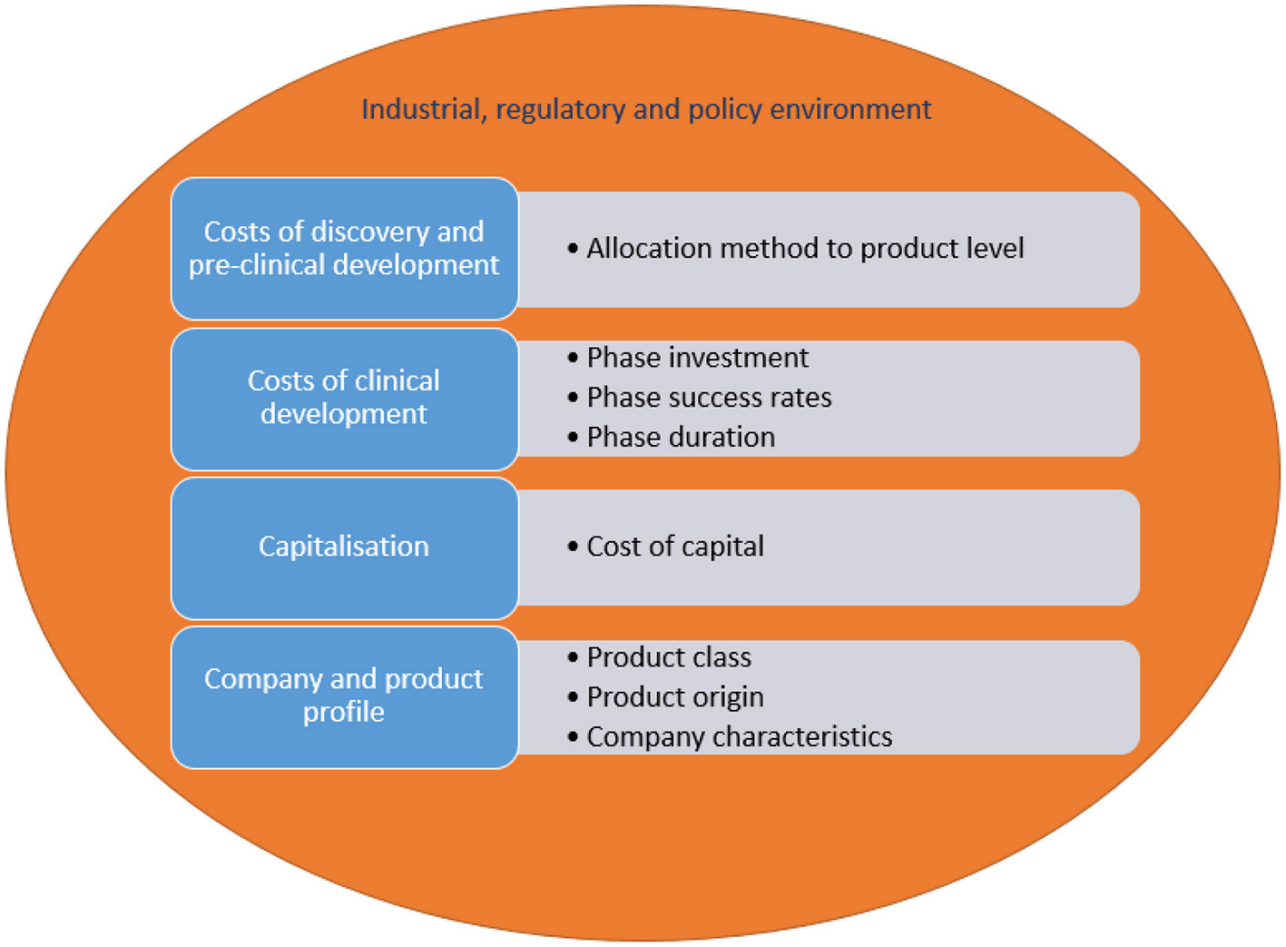
Drivers of R&D costs of new medicines and how these are impacted.

Figure 4 lists the various drivers of R&D costs of new medicines and how these are impacted. Costs relate to discovery and preclinical development, and clinical development. Data on costs of discovery and pre-clinical development are generally not available for a specific medicine, implying that broad company-level data need to be allocated to individual medicines. Different allocation methods have an impact on resulting cost estimates. An estimate of clinical development costs is generated by taking account of the size of investment in the different development phases (i.e., phase I trials, phase II trials, phase III trials, marketing authorisation), phase success rates and duration. Each of these factors are determinants of R&D costs.

In addition, R&D costs need to be “capitalised”. This implies that the estimate is adjusted using a cost of capital, which reflects the return required for an investment today that generates revenues in the future. Capitalisation has a considerable impact: the cost of capital made up 33-51% of total costs per new medicine in four studies (1, 2, 11, 13). The low estimate of costs per new medicine calculated in (14) may derive, amongst other things, from the fact that this study applied a cost of capital of 7%, whereas other studies used higher rates of 10.5%-14.4% (1, 2, 11–13, 15).

Furthermore, the literature indicates that R&D costs per new medicine depend on the product class, whether the medicine is developed in-house or is acquired, and company characteristics (2).

Finally, the broader industrial, regulatory and policy environment influences R&D costs of new medicines. For instance, the literature generates pre-tax estimates and, hence, does not consider deductibility of R&D expenditure for taxation purposes (17). Cost estimates reflect private sector investment in R&D of new medicines and do not account for the public sector contribution (to basic research and other R&D stages). Also, specific policies targeting for example orphan medicines in the United States, Japan and the European Union (such as incentives, expedited review by marketing authorisation authorities, a guaranteed market exclusivity period) support R&D and market access of these products and, hence, affect their costs.

Although a few studies break down R&D costs of new medicines into costs associated with different drivers (2, 11), there is a need to systematically provide estimates of individual cost components in future studies with a view to examine the relative importance of cost components and how their importance evolves over time. Such information could generate insight into R&D productivity and how this could potentially be improved. For instance, a recent literature review has synthesised data about success rates and duration of the different phases of clinical development of new medicines (18). Building on such knowledge, it has been suggested that improving R&D success rates originate from increased validation of drug targets in preclinical research, use of biomarkers to identify subgroups of patients, and the more rapid termination of unpromising medicine candidates (19).

## Conclusion

Estimates of R&D costs per new medicine derived from the peer-reviewed and grey literature vary between US$944m and US$2,826m (adjusted to 2019 prices). The evidence also points toward an increase in these costs over time. Any cost estimate needs to be interpreted in the context of and is specific to the underlying methodology, sample and time period of the study. Estimates are also dynamic: given that the biopharmaceutical industry and the broader environment continuously evolve, the costs to research and develop a new medicine change. Future research would benefit from greater accessibility and transparency of R&D cost data and from improved methodologies to attribute costs of discovery and pre-clinical development to individual medicines.

## Author Contributions

SS developed the idea and design of this study, carried out the literature review, and wrote the manuscript. IH critically reviewed the manuscript. Both authors contributed to the article and approved the submitted version.

## Funding

This research was funded by the European Federation of Pharmaceutical Industries and Associations (EFPIA). The sponsor had no involvement in study design; in the collection, analysis and interpretation of data; in the writing of the paper; and in the decision to submit the paper for publication.

## Conflict of Interest

The authors declare that the research was conducted in the absence of any commercial or financial relationships that could be construed as a potential conflict of interest.

## Publisher's Note

All claims expressed in this article are solely those of the authors and do not necessarily represent those of their affiliated organizations, or those of the publisher, the editors and the reviewers. Any product that may be evaluated in this article, or claim that may be made by its manufacturer, is not guaranteed or endorsed by the publisher.
