# Supplementary material for: R&D Costs of New Medicines: A Landscape Analysis
Source: Front Med (Lausanne). 2021 Oct 26;8:760762. doi: 10.3389/fmed.2021.760762 (PMC8576181; doi:10.3389/fmed.2021.760762)
Supplement: Supplementary file 1 [file Table_1.DOCX]

Supplementary Material

**Data extraction sheets on R&D costs of a new medicine**

| DiMasi JA, Hansen RW, Grabowski HG. The price of innovation: new estimates of drug development costs. J Health Econ. 2003;22(2):151-85. | |
| --- | --- |
| Objective | To calculate the mean pre-tax R&D costs per new successful medicine. |
| Design | Random sample of 68 in-house developed medicines (61 chemical medicines, 6 biologic medicines, 1 vaccine) developed by 10 multinational pharmaceutical companies.  Data were derived from confidential company survey and drug information database.  Sample medicines gained marketing authorisation from Food and Drug Administration between 1990 and 2001.  R&D encompassed research, development and marketing authorisation. |
| Results | Mean capitalised R&D costs per new successful medicine amounted to $802m (price year: 2000). When costs of post-marketing authorization R&D of $95m were included, mean capitalised R&D costs per new successful medicine increased to $897m.  Costs broken down by R&D phase were $335m for discovery and preclinical development and $467m for clinical development. The cost of capital made up 50% of total R&D costs per new successful medicine.  Capitalised R&D costs per new successful medicine were sensitive to changes in cost of capital and clinical study success rate. |
| Conclusions | Analyses of independent data validated study results.  The evolution in R&D costs per new successful medicine over time is likely to depend on factors such as the cost of capital, clinical study success rates, development times, and technological advances in discovery and development. |
| Comments | Sample of medicines and pharmaceutical companies seemed to be representative in terms of disease areas and pharmaceutical R&D expenditure, respectively.  Study did not have access to cost data for each R&D phase of each medicine.  Small sample of medicines.  It is not possible to validate confidential cost data provided by surveyed companies.  It has been argued that this analysis over-estimates costs because it accounts for the cost of capital, it does not consider deductibility of R&D expenditure for taxation purposes, and it is restricted to in-house developed medicines (Light and Lexchin, 2012). |

| Vernon JA, Golec JH, Dimasi JA. Drug development costs when financial risk is measured using the Fama-French three-factor model. Health Econ. 2010;19(8):1002-5. | |
| --- | --- |
| Objective | To update the mean pre-tax R&D costs per new successful medicine from (DiMasi et al., 2003) by using an improved method to estimate the cost of capital. |
| Design | This study applied same methodology as (DiMasi et al., 2003), except for the calculation of the cost of capital.  Cost of capital was 11% in (DiMasi et al., 2003) and 14.36% in this study. |
| Results | Mean capitalised R&D costs per new successful medicine amounted to $992m (price year: 2000). |
| Conclusions | The estimate of mean R&D costs per new successful medicine is sensitive to changes in the cost of capital. |

| Adams CP, Brantner VV. Estimating the cost of new drug development: is it really 802 million dollars? Health Aff (Millwood). 2006;25(2):420-8. | |
| --- | --- |
| Objective | To validate estimate of mean pre-tax R&D costs per new successful medicine from (DiMasi et al., 2003). |
| Design | Analysis combined R&D phase cost data from (DiMasi et al., 2003) with data on success rate and duration of R&D phases from a public drug information database.  Sample medicines that entered phase I-III clinical trial between 1989 and 2002. |
| Results | Mean capitalised R&D costs per new successful medicine amounted to $868m (price year: 2000).  Costs broken down by R&D phase were $381m for discovery and preclinical development and $487 for clinical development.  Capitalised R&D costs per new successful medicine varied between companies and between therapeutic categories due to differences in success rate and duration of R&D phases. |
| Conclusions | This analysis validated study results from (DiMasi et al., 2003). Differences in mean pre-tax R&D costs per new successful medicine between (DiMasi et al., 2003) and this analysis originated from variation in success rate and duration of R&D phases.  There is no single estimate of mean pre-tax R&D costs per new successful medicine, but estimates depend on a number of factors such as company and therapeutic category. |
| Comments | Data included in public database used in this analysis may be less valid than data used in (DiMasi et al., 2003) (e.g. fewer medicines in early phase clinical trials), but related to more medicines under development. |

| Adams CP, Brantner VV. Spending on new drug development. Health Econ. 2010;19(2):130-41. | |
| --- | --- |
| Objective | To validate estimate of mean pre-tax R&D costs per new successful medicine from (DiMasi et al., 2003). |
| Design | Analysis combined phase I-III clinical trial cost data from public database with data on success rates and durations from (Adams and Brantner, 2006).  Clinical trial cost data were derived from examining the relationship between total R&D expenditure of 183 publicly-traded companies and their number of medicines in clinical development between 1989 and 2001. |
| Results | Mean capitalised R&D costs per new successful medicine amounted to $1,214m (price year: 2000).  Capitalised R&D costs per new successful medicine varied between therapeutic categories. |
| Conclusions | Higher mean pre-tax R&D costs per new successful medicine calculated by this analysis than by (DiMasi et al., 2003) originated from higher estimate of phase II clinical trial costs (in addition to differences in success rate and duration of clinical trial phases). |
| Comments | Analysis did not estimate costs of pre-clinical development.  Analysis measured costs of developing an additional medicine instead of costs of the average medicine. |

| DiMasi JA, Grabowski HG, Vernon J. R&D costs and returns by therapeutic category. Drug Information Journal. 2004;38:211-23. | |
| --- | --- |
| Objective | To calculate the mean pre-tax clinical development costs per new successful medicine for several product classes. |
| Design | Sub-sample of (DiMasi et al., 2003) consisting of following product classes: 10 analgesic/anesthetic medicines, 9 anti-infective medicines, 12 cardiovascular medicines, 13 central nervous system medicines.  Data were derived from confidential company survey and drug information database.  R&D encompassed research, development and marketing authorisation. |
| Results | Mean capitalised clinical development costs per new successful medicine amounted to $375m for analgesic/anesthetic medicines, $492m for anti-infective medicines, $460m for cardiovascular medicines, $527m for central nervous system medicines (price year: 2000). |
| Conclusions | The mean clinical development costs per new successful medicine vary between product classes. |
| Comments | This study applied same methodology as (DiMasi et al., 2003).  Inclusion of in-house developed medicines only.  Number of medicines in each product class was limited. |

| DiMasi JA, Grabowski HG. The cost of biopharmaceutical R&D: is biotech different? Manage Decis Econ. 2007;28:469-79. | |
| --- | --- |
| Objective | To calculate the mean pre-tax R&D costs per new successful medicine developed by biotechnology companies. |
| Design | Sample of 9 recombinant proteins and 8 monoclonal antibodies developed by 4 companies.  Data were derived from confidential company survey and drug information database.  R&D encompassed research, development and marketing authorisation. |
| Results | Mean capitalised R&D costs per new successful medicine developed by biotechnology companies amounted to $1,241m (price year: 2005). Costs broken down by R&D phase were $615m for discovery and preclinical development and $626 for clinical development.  This compared with mean capitalised R&D costs per new successful medicine developed by pharmaceutical companies of $1,318m (= adjusted values based on (DiMasi et al., 2003)). Costs broken down by R&D phase were $439m for discovery and preclinical development and $879m for clinical development. |
| Conclusions | Capitalised R&D costs per new successful medicine are similar for biotechnology companies and pharmaceutical companies. |
| Comments | This study applied same methodology as (DiMasi et al., 2003).  Small sample of medicines developed by biotechnology companies.  Care needs to be taken when comparing cost estimates of medicines developed by biotechnology companies and by pharmaceutical companies given that there were differences in disease areas targeted by the two samples of medicines and given that the biotechnology data were more recent than the pharmaceutical data. |

| Paul SM, Mytelka DS, Dunwiddie CT, Persinger CC, Munos BH, Lindborg SR, et al. How to improve R&D productivity: the pharmaceutical industry's grand challenge. Nat Rev Drug Discov. 2010;9(3):203-14. | |
| --- | --- |
| Objective | To calculate the mean pre-tax R&D costs per new successful medicine. |
| Design | This study applied similar methodology, but some different parameter values (specifically with respect to clinical study success rates) as (DiMasi et al., 2003).  Data were derived from 14 large pharmaceutical companies and from previous studies.  R&D encompassed research, development, marketing authorization and launch. |
| Results | Mean capitalised R&D costs per new successful medicine amounted to $1,778m (price year: 2008).  Costs broken down by R&D phase were $824m for discovery and preclinical development and $954m for clinical development. The cost of capital made up 51% of total R&D costs per new successful medicine.  Capitalised R&D costs per new successful medicine were sensitive to changes in clinical study success rates and R&D cycle times. |
| Conclusions | Attrition during clinical development is a significant driver of R&D costs per new successful medicine. |
| Comments | In contrast to (DiMasi et al., 2003), this study excluded costs that are not associated with medicines (e.g. overhead costs).  This study did not consider costs of exploratory discovery research. |

| Mestre-Ferrandiz J, Sussex J, Towse A. The R&D cost of a new medicine. Office of Health Economics; 2012. | |
| --- | --- |
| Objective | To calculate the mean R&D costs per new successful medicine. |
| Design | Sample of approximately 100 medicines from 16 multinational pharmaceutical companies.  Data were derived from confidential company surveys.  R&D encompassed research, development, marketing authorisation and launch. |
| Results | Mean capitalised R&D costs per new successful medicine amounted to $1,506m (price year: 2011). Costs broken down by R&D phase were $207m for discovery, $468m for clinical phase I, $502m for clinical phase II, $294m for clinical phase III, $35m for marketing authorisation and launch. The cost of capital made up 33% of total R&D costs per new successful medicine. |
| Conclusions | The literature shows that R&D costs per new medicine are increasing over time as a result of higher out-of-pocket development costs, falling clinical study success rates, and an increase in the cost of capital. |
| Comments | Few details were provided of data sources (i.e. surveys of pharmaceutical companies) used to inform cost calculations.  Data about costs of clinical phases related to few medicines. |

| DiMasi JA, Grabowski HG, Hansen RW. Innovation in the pharmaceutical industry: New estimates of R&D costs. J Health Econ. 2016;47:20-33. | |
| --- | --- |
| Objective | To calculate the mean pre-tax R&D costs per new successful medicine. |
| Design | This study applied similar methodology as (DiMasi et al., 2003).  Random sample of 106 in-house developed medicines (87 chemical medicines, 19 biologic medicines) developed by 10 multinational pharmaceutical companies.  Data were derived from confidential company survey and drug information database.  Human testing for sample medicines took place between 1995 and 2007.  R&D encompassed research, development and marketing authorisation. |
| Results | Mean capitalised R&D costs per new successful medicine amounted to $2,558m (price year: 2013). When costs of post-marketing authorization R&D of $312m were included, mean capitalised R&D costs per new successful medicine increased to $2,870m.  Costs broken down by R&D phase were $1,098m for discovery and preclinical development and $1,460m for clinical development. The cost of capital made up 45% of total R&D costs per new successful medicine.  Capitalised R&D costs per new successful medicine were sensitive to changes in clinical development costs and clinical study success rate. |
| Conclusions | Analyses of independent data validated study results.  Mean R&D costs per new successful medicine seem to have increased over time. |
| Comments | Sample of medicines seemed to be representative in terms of disease areas, medicine type (chemical or biologic medicine) and development origin (in-house development or acquisition).  Study did not have access to cost data for each R&D phase of each medicine.  Sample of in-house developed medicines. |

| Prasad V, Mailankody S. Research and Development Spending to Bring a Single Cancer Drug to Market and Revenues After Approval. JAMA Intern Med. 2017;177(11):1569-75. | |
| --- | --- |
| Objective | To calculate the mean pre-tax R&D costs per new successful oncology medicine. |
| Design | Sample of 10 medicines from 10 pharmaceutical companies. Sample consisted of 5 in-house developed and 5 acquired medicines. These medicines were approved by Food and Drug Administration between March 2007 and October 2015. Nine medicines had orphan drug designation.  Data were derived from public sources and the literature.  R&D encompassed research, development and marketing authorisation. |
| Results | Mean capitalised R&D costs per new successful oncology medicine amounted to $906m (price year: 2017).  Mean capitalised R&D costs were significantly higher for in-house developed medicines than for acquired medicines. |
| Conclusions | This study generates an R&D cost estimate that can be replicated. |
| Comments | Specific sample of small publicly traded pharmaceutical companies that have 1 approved oncology medicine.  Small sample of oncology medicines with orphan drug designation. Sample was representative of approved oncology medicines.  This study may under-estimate R&D costs of failed medicines.  Methodology did not conform to accounting and finance standards and is likely to have under-estimated R&D costs (Ledley, 2018). |

| Wouters OJ, McKee M, Luyten J. Estimated Research and Development Investment Needed to Bring a New Medicine to Market, 2009-2018. JAMA. 2020;323(9):844-53. | |
| --- | --- |
| Objective | To calculate the mean pre-tax R&D costs per new successful medicine. |
| Design | Sample of 63 medicines developed by 47 companies and approved by Food and Drug Administration between 2009 and 2018. Sample consisted of in-house developed and acquired medicines.  Data were derived from public sources and the literature.  R&D encompassed research, development and marketing authorisation. |
| Results | Mean capitalised R&D costs per new successful medicine amounted to $1,336m (price year: 2018). When limiting the sample to 23 medicines for which high-quality data were available, mean capitalised R&D costs per new successful medicine amounted to $1,143m.  Broken down by product class, mean capitalised R&D costs per new successful medicine varied between $1,077m for central nervous system medicines and $4,461m for antineoplastic and immunomodulating medicines.  Capitalised R&D costs per new successful medicine were sensitive to changes in clinical study success rates, preclinical development expenditure, and cost of capital. |
| Conclusions | This study generates an estimate of R&D costs per new successful medicine derived from public data.  R&D costs per new successful medicine varied between product classes. |
| Comments | Selection bias as orphan medicines, medicines in some disease areas, first-in-class medicines, medicines with expedited marketing authorisation, medicines approved between 2014 and 2018 were over-sampled. Also, private US pharmaceutical companies, foreign companies listed on stock exchange outside US, companies reporting R&D expenditure across medicines or disease areas were excluded. The authors acknowledge that smaller companies were over-sampled.  This study included costs that are not associated with medicines (e.g. overhead costs).  A comparative analysis of cost estimates is inhibited by differences in accounting policies and data reporting between companies.  The authors state that variation in medicine sampling and costing methodology, and lack of public data may explain why their cost estimate differed from those of previous studies. |

**Bibliography**

Adams, C.P., and Brantner, V.V. (2006). Estimating the cost of new drug development: is it really 802 million dollars? Health Aff (Millwood) 25(2), 420-428. doi: 10.1377/hlthaff.25.2.420.

Adams, C.P., and Brantner, V.V. (2010). Spending on new drug development. Health Econ 19(2), 130-141. doi: 10.1002/hec.1454.

DiMasi, J.A., and Grabowski, H.G. (2007). The cost of biopharmaceutical R&D: is biotech different? Manage Decis Econ 28, 469-479.

DiMasi, J.A., Grabowski, H.G., and Hansen, R.W. (2016). Innovation in the pharmaceutical industry: New estimates of R&D costs. J Health Econ 47, 20-33. doi: 10.1016/j.jhealeco.2016.01.012.

DiMasi, J.A., Grabowski, H.G., and Vernon, J. (2004). R&D costs and returns by therapeutic category. Drug Information Journal 38, 211-223.

DiMasi, J.A., Hansen, R.W., and Grabowski, H.G. (2003). The price of innovation: new estimates of drug development costs. J Health Econ 22(2), 151-185. doi: 10.1016/S0167-6296(02)00126-1.

Ledley, F.D. (2018). Methods Used to Assess Pharmaceutical Research and Development Costs. JAMA Intern Med 178(4), 589. doi: 10.1001/jamainternmed.2018.0110.

Light, D.W., and Lexchin, J.R. (2012). Pharmaceutical research and development: what do we get for all that money? BMJ 345, e4348. doi: 10.1136/bmj.e4348.

Mestre-Ferrandiz, J., Sussex, J., and Towse, A. (2012). "The R&D cost of a new medicine". Office of Health Economics).

Paul, S.M., Mytelka, D.S., Dunwiddie, C.T., Persinger, C.C., Munos, B.H., Lindborg, S.R., et al. (2010). How to improve R&D productivity: the pharmaceutical industry's grand challenge. Nat Rev Drug Discov 9(3), 203-214. doi: 10.1038/nrd3078.

Prasad, V., and Mailankody, S. (2017). Research and development spending to bring a single cancer drug to market and revenues after approval. JAMA Intern Med 177(11), 1569-1575. doi: 10.1001/jamainternmed.2017.3601.

Vernon, J.A., Golec, J.H., and Dimasi, J.A. (2010). Drug development costs when financial risk is measured using the Fama-French three-factor model. Health Econ 19(8), 1002-1005. doi: 10.1002/hec.1538.

Wouters, O.J., McKee, M., and Luyten, J. (2020). Estimated research and development investment needed to bring a new medicine to market, 2009-2018. JAMA 323(9), 844-853. doi: 10.1001/jama.2020.1166.
